# Mental Health and Involuntary Retirement from Sports Post-Musculoskeletal Injury in Adult Athletes: a Systematic Review

**DOI:** 10.1007/s12178-023-09830-6

**Published:** 2023-04-04

**Authors:** Kira Furie, Anna L. Park, Stephanie E. Wong

**Affiliations:** 1 University of California San Francisco School of Medicine, San Francisco, CA 94143 USA; 2 Department of Orthopaedic Surgery, University of California San Francisco, San Francisco, CA 94143 USA; 3 San Francisco, CA USA

**Keywords:** Mental health, Musculoskeletal injury, Athlete, Retirement from sport

## Abstract

**Purpose of Review:**

The psychological aspects of musculoskeletal injury are often overlooked in the rehabilitation process. This review examines the effects of musculoskeletal injury on mental health in adult athletes and identifies themes to guide further research.

**Recent Findings:**

Athletes are at risk for mental health struggles due to high athletic identity and identity foreclosure. Injured athletes have specifically been shown to have higher rates of anxiety and depression when compared to the general population. There is a lack of intervention-based research on the psychological well-being of athletes, and there are no systematic reviews synthesizing the impact of musculoskeletal injury on the mental health of adult athletes across a variety of sports.

**Summary:**

Across professional, college-level, and amateur athletes, musculoskeletal injury is associated with worse mental health scores, including higher distress, higher anxiety and depression, lower social functioning, and lower health-related quality of life. For adults, involuntary retirement from sports due to musculoskeletal injury is a common theme associated with increased psychological distress, anxiety, and depression. In the reviewed literature, 22 unique mental health and 12 distinct physical health screening tools were used. Two articles studied interventions addressing mental health post-injury. Further research using an integrated physical and psychological approach to recovery is warranted and may improve mental and physical outcomes for injured athletes.

## Introduction

Musculoskeletal injury in adult athletes is extremely common. Amongst a variety of sports and levels of competition with imaging confirmation and/or a positive physical exam by an orthopedist, musculoskeletal injuries have been reported at a prevalence of 77% [1]. Similarly, 78% of collegiate athletes experience musculoskeletal injury [2].

Athletes as a population are particularly vulnerable to mental health struggles due to high athletic identity, identity foreclosure, and a social world that often leads to a loss of personal autonomy [3, 4]. Athlete identity refers to the strength and exclusivity to which an individual identifies with their role as an athlete [5]. Athletic identity foreclosure combines athlete identity with identity foreclosure to describe a state in which athletes are highly committed to their role without having explored occupational or ideological alternatives [6].

While elite athletes have been shown to have a similar prevalence of mental disorders compared to the general population [4, 7], injured athletes have been shown to have higher levels of anxiety and depression [7]. There are relatively few methodologically rigorous studies on mental health in athletes compared to those focused on physical health, and there is a lack of intervention-based research on the psychological well-being of athletes [4].

To create an effective intervention, a clear picture of the problem is needed [8]; however, there are no reviews to date synthesizing the impact of musculoskeletal injury on the mental health of adult athletes across a variety of sports. The purpose of this review is to assess the existing literature on mental health post-injury in adult athletes and to identify themes to guide further research.

## Methods

### Search Strategy

Two online databases (PubMed and PsycNet) were searched for literature addressing mental health after injury in adult athletes. Search terms included ((athlete OR sports OR athletic) AND (mental health AND (injury) NOT (pediatric OR adolescent OR child)).

### Study Screening

Our search yielded 1071 articles, 61 of which were duplicates across the two databases. Titles and abstracts were sequentially reviewed by two authors (KF, AP), and disagreements were resolved by discussion between these two authors after abstract screening.

### Assessment of Study Eligibility

Studies that discussed the impacts of musculoskeletal injury on mental health in adult athletes were included. Studies focused on traumatic brain injury (TBI) or concussion were excluded due to potentially different mechanisms affecting brain injury versus musculoskeletal injury. Studies on pediatric populations and studies that did not discuss mental health specifically after injury were excluded. Letters to the editor, dissertations, books, and book chapters were excluded.

### Data Extraction

The measures used to assess physical and mental health were extracted and their frequency of use across the 31 articles was recorded. Key themes that emerged across college-level, professional, and mixed levels of athletes were synthesized and recorded.

## Results

### Study Characteristics

**Fig. 1:**
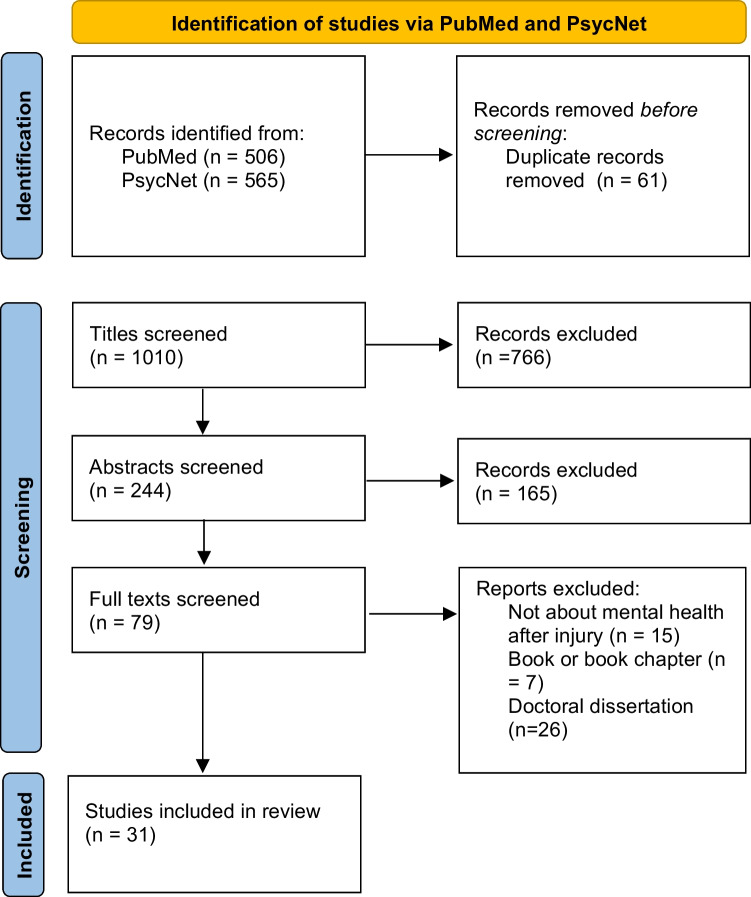
PRISMA diagram: title, abstract, and full text screening yielded 31 studies

After title and abstract screening, 79 articles were included for full text review. Upon full text review using the exclusion criteria, 48 additional articles were excluded based on relevance and type of publication (Fig. 1).

**Fig. 2:**
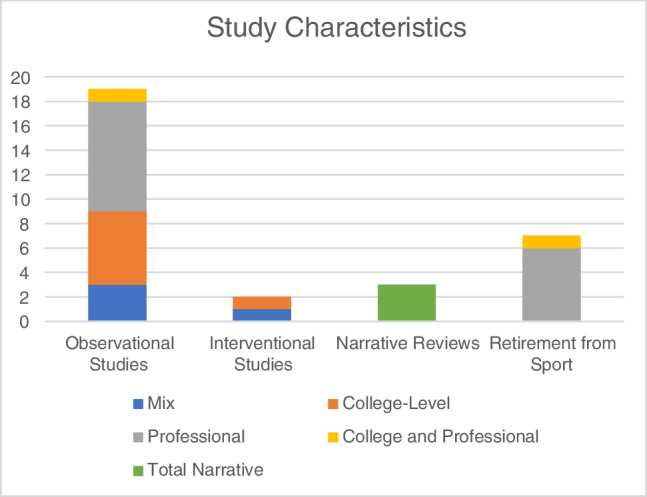
Studies included in review by category

Of the 31 included articles, there were 19 observational studies. Of these 19 studies, 3 were on a mixed of level of athletes, 6 on college athletes, 9 on professional athletes, and 1 on both college and professional athletes. There were 2 interventional studies—1 in college athletes and 1 on mixed levels of athletes. There were 3 narrative reviews and 7 studies which discussed retirement from sport (Fig. 2).

There were 22 unique mental health (Table 1) and 12 distinct physical health measures (Table 2) used across the 31 included articles. Of the mental health measures, there were 5 on anxiety and depression combined, 4 on depression/mood disorders, 3 on quality of life, 2 on mindfulness, 2 on mental well-being, 2 on substance use, 1 on anxiety/stress disorders, 1 on athletic identity, 1 on eating disorders, and 1 on psychosis.

**Table 1:** Mental health measures and citations using each measure

Mental health measure	Number of papers using measure	Citation(s)
Anxiety/stress disorders		
Social Athletic Readjustment Rating Scale (SARRS)	2	Kiliç et al., 2018 [9, 10] Schuring et al., 2017 [10]
Anxiety and depression combined		
12-Item General Health Questionnaire (GHQ-12)	4	Kilic et al., 2019 [11••] Kiliç et al., 2018 [9] Brown et al., 2017 [12] Schuring et al., 2017 [10]
Diagnostic and Statistical Manual of Mental Disorders, 5^th^ Edition (DSM-5)	3	Li et al., 2021 [13] Aron et al., 2019 [14] Rice et al., 2019 [15]
Four-Dimensional Symptom Questionnaire (4DSQ)	3	Kilic et al., 2019 [11••] Brown et al., 2017 [12] Schuring et al., 2017 [10]
Depression Anxiety Stress Scales (DASS)	1	Mohammed et al., 2018 [16]
Patient Health Questionnaire-4 (PHQ-4)	1	Moesch et al., 2020 [17]
Athletic Identity		
Athletic Identity Measurement Scale (AIMS)	2	Sanders and Stevinson, 2017 [18] Brewer et al., 1995 [19]
Depression/mood disorders		
Profile of Mood States (POMS)	2	Mohammed et al., 2018 [16] Brewer et al., 1995 [19]
Beck Depression Inventory (BDI)	1	Brewer et al., 1995 [19]
Center for Epidemiological Studies-Depression Scale (CES-D)	1	Vergeer, 2006 [20]
Short Depression-Happiness Scale (SDHS)	1	Sanders and Stevinson, 2017 [18]
Eating disorders		
Eating Disorder Screen for Primary Care (EDS-PC)	1	Vergeer, 2006 [20]
Mindfulness		
Five Facet Mindfulness Questionnaire (FFMQ)	1	Moesch et al., 2020 [17]
Mindful Attention Awareness Scale (MAAS)	1	Mohammed et al., 2018 [16]
Mental well-being		
Warwick-Edinburgh Mental Wellbeing Scale (WEMWBS)	1	Abbott et al., 2019 [21]
World Health Organization- Five Well-Being Index (WHO-5)	1	Moesch et al., 2020 [17]
Psychosis		
Prodromal Questionnaire (PQ)	1	Brewer et al., 1995 [19]
Quality of life		
Patient-Reported Outcomes Measurement Information System- Short Form (PROMIS-SF)	6	Cross et al., 2022 [22] DeFreese et al., 2022 [23] Schuring et al., 2019 [10] Kilic et al., 2019 [11••] Kiliç et al., 2018 [9] Brown et al., 2017 [12]
36-Item Short Form Survey (SF-36)	6	Filan et al., 2021 [24] Kerr et al., 2021 [25] Pehlivanoglu et al., 2021 [26] Cowee and Simon, 2019 [27] Moreira et al., 2016 [28] Andrew et al., 2012[29]
12-Item Short Form Survey (SF-12)	1	Marshall et al., 2020 [30]
Substance use		
Alcohol Use Disorders Identification Test- Concise (AUDIT-C)	4	Kilic et al., 2019 [11••] Kiliç et al., 2018 [9] Brown et al., 2017 [12] Schuring et al., 2017 [10]
Substance Abuse Assessment Questionnaire (SAAQ)	1	Moesch et al., 2020 [17]

**Table 2:** Physical health measures and citations using each measure

Physical health measure	Number of papers using measure	Citation(s)
36-Item Short Form Survey (SF-36)	6	Filan et al., 2021 [24] Kerr et al., 2021 [25] Pehlivanoglu et al., 2021 [26] Cowee and Simon, 2019 [27] Moreira et al., 2016 [28] Andrew et al., 2012[29]
International Physical Activity Questionnaire (IPAQ)	2	Moreira et al., 2016 [28] Andrew et al., 2012 [29]
Cold Pressor Test (CPT)	1	Mohammed et al., 2018 [16]
Disablement in the Physically Active Scale (DPA)	1	Cross et al., 2022 [22]
Foot and Ankle Ability Measure (FAAM)	1	Marshall et al., 2020 [30]
Glasgow Outcome Scale- Extended (GOSE)	1	Andrew et al., 2012 [29]
Godin-Shephard Leisure Time Physical Activity Questionnaire (GSLTPAQ)	1	Cross et al., 2022 [22]
Modified Harris Hip Score (mHHS)	1	Filan et al., 2021 [24]
Pain Intensity Numerical Rating Scale	1	Sanders and Stevinson, 2017 [18]
Visual Analog Scale (VAS)	1	Mohammed et al., 2018 [16]
Western Ontario and McMaster Universities Osteoarthritis Index (WOMAC)	1	Filan et al., 2021 [24]
12-Item Short Form Survey (SF-12)	1	Marshall et al., 2020 [30]

### Observational Studies

#### Mixed Performance Levels

Sports injury is associated with worse mental health and social functioning in basketball players [28]. A systematic review on mental health and injury in football players suggests an association between injury and depression, although one study included in the review found no association between depression and injury [31••]. One study investigated surgical intervention for injury, finding that arthroscopic correction of femoroacetabular impingement in male athletes was shown to improve physical health outcomes but did not have an effect on mental health [24].

#### Collegiate

In college athletes, musculoskeletal injury is associated with higher anxiety and is a significant risk factor for generalized anxiety disorder [13, 15]. Injury has also been associated with increased risk of depression in athletes [32]. Women were found to be more susceptible to depressive symptoms compared to men regardless of injury status [20]. With ankle injury specifically, increased injury severity is correlated with worse mental health outcomes in comparison to mild or no injury [30]. By contrast, some studies including knee, ankle, soft tissue, and various other injury types found that injury status did not correlate to worse mental health scores [22, 27].

#### Professional

Of the 10 observational studies discussing professional athletes, there are 6 on soccer players, 1 about cricketers, 1 on rugby players, 1 on National Football League (NFL), and 1 on professional athletes in general. Across a variety of professional athletes, musculoskeletal injury is associated with higher anxiety [15]. In soccer players, severe musculoskeletal time-loss injury at baseline, defined as an injury that occurred during team activities that led to an absence from training or a match for > 28 days, is associated with a relative risk of 6.9 of developing distress by 12-month follow-up [9] and mental well-being scores when injured are lower compared to when healthy [21]. Severe musculoskeletal time-loss injuries in male soccer players, such as ACL tears and cartilage tears, are also associated with mental health difficulty and feelings of worthlessness. Injury is perceived as a threat to survival as a professional soccer player, and the psychological distress experienced is associated with breakdown of athlete identity [33, 34]. In male European professional soccer players, the number of severe musculoskeletal injuries attained and surgeries undergone during their careers is positively correlated with symptoms of distress, sleep disorders, adverse alcohol behavior, and adverse nutritional behavior [35]. Interviews with professional soccer players revealed that injury contributed to the psychological distress affecting daily functioning and relationships. Injury education was important for reducing stress. Negative thoughts and emotions affecting self-image were barriers to recovery and return to play, and having emotional and professional psychological support was crucial to recovery and return to play [34]. Sports psychologists reported that sports injuries in professional soccer players are associated with long-term psychological and behavioral consequences including depression, loss of identity, loss of future, emotional disturbance, gambling, substance abuse, sport avoidance, and isolation [36•]. In current and retired professional cricketers, surgery is associated with increased risk of common mental disorders, defined as anxiety/depression, sleep disturbance, and adverse alcohol use [10]. NFL players with head/neck/face and lower leg/foot/ankle injuries that are still affecting them at the time of assessment have lower mental health scores compared to the group without any injuries [25]. Male professional rugby players who sustained severe injury within 12 months of baseline assessment were more likely to develop symptoms of anxiety or depression (odds ratio 1.5) [11••].

### Interventional Studies

#### Mixed Performance Levels

Elite and amateur athletes who underwent a mindfulness and acceptance-based intervention showed increases in well-being and nonreactivity [17].

#### Collegiate

In athletes with an injury requiring them to have at least 3 months out of sports, the group who completed a modified Mindfulness-Based Stress Reduction (MBSR) course had lower anxiety and higher mindfulness attention awareness scores [16].

### Narrative Reviews

Symptoms of acute stress disorder are seen as soon as 2 days after traumatic physical injury in 23–45% of athletes, with 50% of these cases later meeting criteria for post-traumatic stress disorder (PTSD) [14]. Problematic emotional reactions to injury include sleep disturbance, irritability, and persistent alterations in appetite. Worsening symptoms after injury include disordered eating, depression, apathy, and alienation. Expressive symptoms in response to injury include pain behaviors, rage, emotional outbursts, and substance abuse [37]. Imagery, relaxation, goal setting, brief counseling, and social support from coaches and teammates were found to be helpful in athletes’ recovery from injury [38].

### Retirement from Sport

Psychological distress, waning physical fitness, loss of athletic identity, career transition, potential for substance misuse, disordered eating, and sleep disturbances are all associated with retirement from sport in collegiate and professional athletes [39•]. In former NFL players, involuntary discontinuation from sports and no transition plan are associated with higher depressive and anxiety symptom severity [23]. Players who are forced to retire from professional rugby are twice as likely to report symptoms of distress compared to those who retired voluntarily [12].

In professional cricketers, current players have a higher incidence of mental disorders compared to retired players [10]. In professional soccer players who meet the clinical cutoff for depression, depression is independently associated with retirement due to injury, higher pain levels, and increased athletic identity [18]. In professional soccer players, transition out of sports is associated with feelings of loneliness, sadness, failure, fear, and loss of purpose and identity [33].

## Discussion

This systematic review represents a comprehensive synthesis of literature published on mental health after injury in adult athletes along with themes that emerged in the review process, most notably involuntary retirement from sport secondary to injury. The majority (6 out of 7) of the articles discussing retirement from sport were on various types of professional athletes.

Across collegiate, professional, and mixed-level studies, injury in adult athletes was associated with higher distress, worse mental health scores, and maladaptive behaviors such as disordered eating and substance abuse.

Of the 31 included articles, 3 discussed surgical intervention for injury while the remaining 28 did not mention surgical intervention specifically. Only one of the studies focused exclusively on female athletes, while 4 studies included only male athletes, despite the finding that female athletes score lower on mental health, social functioning, and general health when compared to male athletes [30]. Additionally, no studies included in this review compared emotional disturbance after injury between levels of athletes; however, increased age has been shown to be negatively associated with post-injury emotional disturbance [19].

Barriers to seeking help for poor mental health after injury include lack of education, stigma, negative attitudes towards help-seeking, and accessibility. Facilitators to seeking help include education, attitudes and actions of others, and accessibility [40]. This suggests that mental health after injury may be best addressed by the care team educating injured athletes on options for mental health care and more broadly by addressing social determinants of health that may limit the accessibility to mental health resources.

Despite the fact that athletic injury is associated with worse mental health, only 2 of the 31 articles discussed interventions. This vulnerable period for athletes represents an opportunity for future interventions to aid the injured athlete in their recovery and/or retirement from sport.

### Suggested Interventions

An integrated approach to rehabilitation is recommended. The Integrated Sports Injury model encompasses (a) the impact of biopsychosocial variables on the stress response and the likelihood of injury onset, (b) the personal and situational influencing factors, and (c) the cognitive, emotional, and behavioral responses to the injury during the rehabilitation process. Imagery, relaxation, goal setting, positive self-talk, coping skills, modeling, psychoeducation, biofeedback, and social support led to positive mood changes, improved self-efficacy, reduced stress and anxiety, improved motivation and satisfaction, more effective pain management, enhanced exercise compliance, and improved rehabilitation [40]. MBSR, Acceptance and Commitment Therapy, and Motivational Enhancement Therapy have been shown to be effective in improving the rehabilitation process [16, 40].

One paper suggests further study on psilocybin-assisted psychotherapy for injured athletes, noting that higher athletic identity is associated with increased depression during the injury rehabilitation period, and psychedelics have been shown to lead to a deeper acceptance and understanding of one’s identity. While psilocybin is currently not approved for use during competition, psilocybin use in psychotherapy has been increasing and gaining traction. Further study is needed to assess psilocybin-assisted psychotherapy’s efficacy, safety, and effects on sport performance [41].

A case study of a gridiron football player in Australia post-ACL injury reported journaling about the injury can decrease stress and mood disturbance via emotional disclosure and cognitive integration to help understanding of injury [42].

In regard to retirement from sport, mental health screening, sports psychology, exit examination with nutritionist or team doctor, education on sleep hygiene and healthy alcohol usage, and formal discussion about the athlete’s relationship with exercise post-retirement are recommended [39•]. The American Society for Sports Medicine 2019 Position Statement recommends that athletic departments, national governing bodies, and professional leagues create comprehensive retirement plans for athletes who are transitioning out of sport [43].

Current initiatives to support athletes in transition out of sport include university-specific pages with information and podcasts interviewing athletes who have had career-ending injuries. The recommendation for comprehensive care to prepare athletes for retirement paired with the limited initiatives that exists demonstrates a gap that future interventions should seek to address.

### Limitations

The majority of the articles in this study focus on collegiate and professional athletes, with little mention of amateur or recreational athletes, or comparison between different levels of competition. In addition, while a variety of sports are included in this review, not all sports are represented. These factors limit the generalizability of the present study.

## Conclusions

In conclusion, review of the literature revealed that musculoskeletal injury and involuntary retirement from sport secondary to injury are associated with worse mental health in adult athletes, which has been shown to improve with mindfulness-based interventions. Further interventions using an integrated physical and psychological approach to recovery are warranted.

## References

[1] Goes RA, Lopes LR, Cossich VRA, de Miranda VAR, Coelho ON, do Carmo Bastos R (2020). Musculoskeletal injuries in athletes from five modalities: a cross-sectional study. BMC Musculoskelet Disord..

[2] Lemoyne J, Poulin C, Richer N, Bussières A (2017). Analyzing injuries among university-level athletes: prevalence, patterns and risk factors. J Can Chiropr Assoc.

[3] Hughes L, Leavey G (2012). Setting the bar: athletes and vulnerability to mental illness. Br J Psychiatry J Ment Sci.

[4] Rice SM, Purcell R, De Silva S, Mawren D, McGorry PD, Parker AG (2016). The Mental Health of Elite Athletes: A Narrative Systematic Review. Sports Med Auckl NZ.

[5] Brewer BW, Petitpas AJ, Van Raalte JL, Maher MT (1993). Identity foreclosure, athletic identity, and college sport participation. Acad Athl J.

[6] Brewer BW, Petitpas AJ (2017). Athletic identity foreclosure. Curr Opin Psychol.

[7] Gulliver A, Griffiths KM, Mackinnon A, Batterham PJ, Stanimirovic R (2015). The mental health of Australian elite athletes. J Sci Med Sport.

[8] O’Cathain A, Croot L, Duncan E, Rousseau N, Sworn K, Turner KM (2019). Guidance on how to develop complex interventions to improve health and healthcare. BMJ Open.

[9] Kiliç Ö, Aoki H, Goedhart E, Hägglund M, Kerkhoffs GMMJ, Kuijer PPFM (2018). Severe musculoskeletal time-loss injuries and symptoms of common mental disorders in professional soccer: a longitudinal analysis of 12-month follow-up data. Knee Surg Sports Traumatol Arthrosc Off J ESSKA.

[10] Schuring N, Kerkhoffs G, Gray J, Gouttebarge V (2017). The mental wellbeing of current and retired professional cricketers: an observational prospective cohort study. Phys Sportsmed.

[11] Kilic Ö, Hopley P, Kerkhoffs GMMJ, Lambert M, Verhagen E, Viljoen W (2019). Impact of concussion and severe musculoskeletal injuries on the onset of mental health symptoms in male professional rugby players: a 12-month study. BMJ Open Sport Exerc Med.

[12] Brown JC, Kerkhoffs G, Lambert MI, Gouttebarge V (2017). Forced Retirement from Professional Rugby Union is Associated with Symptoms of Distress. Int J Sports Med.

[13] Li C, Fan R, Sun J, Li G (2021). Risk and Protective Factors of Generalized Anxiety Disorder of Elite Collegiate Athletes: A Cross-Sectional Study. Front Public Health.

[14] Aron CM, Harvey S, Hainline B, Hitchcock ME, Reardon CL (2019). Post-traumatic stress disorder (PTSD) and other trauma-related mental disorders in elite athletes: a narrative review. Br J Sports Med.

[15] Rice SM, Gwyther K, Santesteban-Echarri O, Baron D, Gorczynski P, Gouttebarge V (2019). Determinants of anxiety in elite athletes: a systematic review and meta-analysis. Br J Sports Med.

[16] Mohammed WA, Pappous A, Sharma D (2018). Effect of Mindfulness Based Stress Reduction (MBSR) in Increasing Pain Tolerance and Improving the Mental Health of Injured Athletes. Front Psychol.

[17] Moesch K, Ivarsson A, Johnson U (2020). “Be Mindful Even Though It Hurts”: A Single-Case Study Testing the Effects of a Mindfulness- and Acceptance-Based Intervention on Injured Athletes’ Mental Health. J Clin Sport Psychol.

[18] Sanders G, Stevinson C (2017). Associations between retirement reasons, chronic pain, athletic identity, and depressive symptoms among former professional footballers. Eur J Sport Sci.

[19] Brewer BW, Linder DE, Phelps CM (1995). Situational correlates of emotional adjustment to athletic injury. Clin J Sport Med Off J Can Acad Sport Med.

[20] Vergeer I (2006). Exploring the mental representation of athletic injury: A longitudinal case study. Psychol Sport Exerc.

[21] Abbott W, Brownlee TE, Harper LD, Naughton RJ, Richardson A, Clifford T (2019). A season long investigation into the effects of injury, match selection and training load on mental wellbeing in professional under 23 soccer players: A team case study. Eur J Sport Sci.

[22] Cross SJ, Gill DL, Brown PK, Reifsteck EJ (2022). Prior Injury, Health-Related Quality of Life, Disablement, and Physical Activity in Former Women’s Soccer Players. J Athl Train.

[23] DeFreese JD, Walton SR, Kerr ZY, Brett BL, Chandran A, Mannix R (2022). Transition-Related Psychosocial Factors and Mental Health Outcomes in Former National Football League Players: An NFL-LONG Study. J Sport Exerc Psychol.

[24] Filan D, Carton P (2021). Chronic Hip Injury Has a Negative Emotional Impact on the Male Athlete With Femoroacetabular Impingement. Arthrosc J Arthrosc Relat Surg Off Publ Arthrosc Assoc N Am Int Arthrosc Assoc.

[25] Kerr ZY, Prim J, DeFreese JD, Thomas LC, Simon JE, Carneiro KA (2021). Musculoskeletal Injury History Is Associated With Lower Physical and Mental Health in a Historical Cohort of Former National Football League Players. J Sport Rehabil.

[26] Pehlivanoglu T, Oltulu I, Erdag Y, Akturk UD, Korkmaz E, Yildirim E (2021). Comparison of clinical and functional outcomes of vertebral body tethering to posterior spinal fusion in patients with adolescent idiopathic scoliosis and evaluation of quality of life: preliminary results. Spine Deform.

[27] Cowee K, Simon JE (2019). A History of Previous Severe Injury and Health-Related Quality of Life Among Former Collegiate Athletes. J Athl Train.

[28] Moreira NB, Mazzardo O, Vagetti GC, De Oliveira V, De Campos W (2016). Quality of life perception of basketball master athletes: association with physical activity level and sports injuries. J Sports Sci.

[29] Andrew NE, Wolfe R, Cameron P, Richardson M, Page R, Bucknill A (2012). Return to pre-injury health status and function 12 months after hospitalisation for sport and active recreation related orthopaedic injury. Inj Prev J Int Soc Child Adolesc Inj Prev.

[30] Marshall AN, Snyder Valier AR, Yanda A, Lam KC (2020). The Impact of a Previous Ankle Injury on Current Health-Related Quality of Life in College Athletes. J Sport Rehabil.

[31] Sarmento H, Frontini R, Marques A, Peralta M, Ordoñez-Saavedra N, Duarte JP (2021). Depressive Symptoms and Burnout in Football Players: A Systematic Review. Brain Sci.

[32] Wolanin A, Gross M, Hong E (2015). Depression in athletes: prevalence and risk factors. Curr Sports Med Rep.

[33] Wood S, Harrison LK, Kucharska J (2017). Male professional footballers’ experiences of mental health difficulties and help-seeking. Phys Sportsmed.

[34] Borg AF, Falzon R, Muscat A (2021). Psychological implications and rehabilitation programmes due to football-related injuries. Couns Psychother Res.

[35] Moesch K, Kenttä G, Kleinert J, Quignon-Fleuret C, Cecil S, Bertollo M (2018). FEPSAC position statement: Mental health disorders in elite athletes and models of service provision. Psychol Sport Exerc.

[36] Gervis M, Pickford H, Hau T (2019). Professional Footballers’ Association Counselors’ Perceptions of the Role Long-Term Injury Plays in Mental Health Issues Presented by Current and Former Players. J Clin Sport Psychol.

[37] Putukian M (2016). The psychological response to injury in student athletes: a narrative review with a focus on mental health. Br J Sports Med.

[38] Wiese-Bjornstal DM (2010). Psychology and socioculture affect injury risk, response, and recovery in high-intensity athletes: a consensus statement. Scand J Med Sci Sports.

[39] Esopenko C, Coury JR, Pieroth EM, Noble JM, Trofa DP, Bottiglieri TS (2020). The Psychological Burden of Retirement from Sport. Curr Sports Med Rep..

[40] O’Keeffe S, NíChéilleachair N, Campbell M, O’Connor S (2022). Barriers and Facilitators to Mental Health Help-Seeking in Elite Gaelic Footballers Post-Injury: A Qualitative Study. Res Q Exerc Sport.

[41] Walton CC, Liknaitzky P (2022). Advancing elite athlete mental health treatment with psychedelic-assisted psychotherapy. J Appl Sport Psychol.

[42] Mankad A, Gordon S, Wallman K (2009). Psycholinguistic Analysis of Emotional Disclosure: A Case Study in Sport Injury. J Clin Sport Psychol.

[43] Chang C, Putukian M, Aerni G, Diamond A, Hong G, Ingram Y (2020). Mental health issues and psychological factors in athletes: detection, management, effect on performance and prevention: American Medical Society for Sports Medicine Position Statement-Executive Summary. Br J Sports Med.

